# Important Roles of Key Genes and Transcription Factors in Flower Color Differences of *Nicotiana alata*

**DOI:** 10.3390/genes12121976

**Published:** 2021-12-10

**Authors:** Yalin Zheng, Yudong Chen, Zhiguo Liu, Hui Wu, Fangchan Jiao, Haiping Xin, Li Zhang, Long Yang

**Affiliations:** 1 College of Plant Protection and Agricultural Big-Data Research Center, Shandong Agricultural University, Tai’an 271018, China; 18864805679@163.com (Y.Z.); sdauzbcyd@163.com (Y.C.); 17863800846@163.com (Z.L.); 18864805709@163.com (H.W.); lilizhang324@163.com (L.Z.); 2 Key Laboratory of Tobacco Biotechnological Breeding, National Tobacco Genetic Engineering Research Center, Yunnan Academy of Tobacco Agricultural Sciences, Kunming 650021, China; jfc99002@163.com; 3 CAS Key Laboratory of Plant Germplasm Enhancement and Specialty Agriculture, Wuhan Botanical Garden, Chinese Academy of Sciences, Wuhan 430074, China; xinhaiping@wbgcas.cn

**Keywords:** *Nicotiana alata*, chlorophyll metabolism pathway, anthocyanin biosynthesis pathway, transcriptome

## Abstract

*Nicotiana alata* is an ornamental horticultural plant with a variety of flower colors and a long flowering period. The genes in four different colored *N. alata* (white, purple, red, and lemon green) were analyzed to explain the differences in flower color using transcriptomes. A total of 32 differential expression genes in the chlorophyll biosynthesis pathway and 41 in the anthocyanin biosynthesis pathway were identified. The enrichment analysis showed that the chlorophyll biosynthesis pathway and anthocyanin biosynthesis pathway play critical roles in the color differences of *N. alata*. The *HEMA* of the chlorophyll biosynthesis pathway was up-regulated in lemon green flowers. Compared with white flowers, in the red and purple flowers, *F3H*, *F3′5′H* and *DFR* were significantly up-regulated, while *FLS* was significantly down-regulated. Seventeen differential expression genes homologous to transcription factor coding genes were obtained, and the homologues of *HY5*, *MYB12, AN1* and *AN4* were also involved in flower color differences. The discovery of these candidate genes related to flower color differences is significant for further research on the flower colors formation mechanism and color improvements of *N. alata*.

## 1. Introduction

*Nicotiana alata* belongs to the *Solanaceae* family, which is used as an ornamental horticultural plant because of its rich and bright colors and long flowering period [1,2]. Flower color is an essential character of ornamental plants. The colors of flowers are mainly determined by types of pigments and their contents, such as chlorophylls and anthocyanins [3,4].

Chlorophylls are photosynthetic pigments commonly found in photosynthetic tissue. The existence of chlorophyll is the main reason for the green appearance of petals. Chlorophylls are present in many flowering plants during the early development stage of petals, and their contents gradually decrease as the petals develop [4]. The chlorophyll metabolism pathway has been studied in petals in many species, such as carnation [5], chrysanthemum [4,6,7] and petunia [8]. 5-Aminolevulinic acid (ALA) biosynthesis is the rate-limiting step of chlorophyll synthesis, and ALA is formed from glutamyl-tRNA by two enzymatic steps. The *HEMA* encodes the glutamyl-tRNA reductase (GluTR), the first ALA synthesis enzyme [9].

Anthocyanins belong to flavonoids, which are water-soluble pigments widely found in plants [10]. They have a variety of chemical structures and usually make plants appear red, blue, and purple [11,12]. Anthocyanin biosynthesis is controlled by early structural genes (*PAL*: phenylalanine ammonia-lyase, *C4H*: cinnamate 4-hydroxylase, *4CL*: 4-coumarate-CoA ligase, *CHS*: chalcone synthase, *CHI*: chalcone isomerase and *F3H*: flavanone 3-hydroxylase) and late structural genes (*F3′H*: flavonoid 3′-hydroxylase, *F3′5′H*: flavonoid 3′,5′-hydroxylase, *FLS*: flavonol synthase, *DFR*: dihydroflavonol 4-reductase, *ANS*: anthocyanidin synthase, *ANR*: anthocyanidin reductase and *UFGT*: UDP-glucose:flavonoid 3-O-glucosyltransferase) [13,14,15]. How genes in the anthocyanin biosynthesis pathway play roles in flower color formation has been studied in *Arabidopsis thaliana*, *Nicotiana tabacum* and other plants [16,17,18,19]. DFR competes with FLS for substrates to convert dihydroflavonols into leucoanthocyanins or flavonol aglycones. That is an important node of the anthocyanin biosynthesis pathway as distinguished from the flavonol biosynthesis pathway [14,20]. Leucoanthocyanins are the precursors of anthocyanin synthesis. In this way, DFR promotes anthocyanins accumulation, thus making the flowers redder or more purple. FLS does the opposite.

In addition, flower color formation is regulated by transcription factors (TFs) [21]. The bZIP, MYB, bHLH and WD40-repeat are major transcription factor families that regulate the anthocyanin biosynthesis pathway [22,23,24,25,26]. The expression of *HY5* in the bZIP family was positively correlated with anthocyanin contents in peony. In the process of flower color change (coral-pink-pale yellow) of peony, its expression increased at first and then decreased [27]. SlMYB12 is the homologue of AtMYB12, which was considered to induce the expression of *CHS* and *FLS* [28]. It promotes FLS to compete for substrates from the anthocyanin biosynthesis pathway, decreasing anthocyanin accumulation and lighter flower color. Studies in petunia showed that AN1 (bHLH family) could activate the expression of *DFR* alone or in cooperation with AN2 (MYB family), and the expression of *AN1* was regulated by AN2 and AN4 (MYB family) in anther [29]. MYB, bHLH and WD40 families could also form MYB-bHLH-WD40 (MBW) protein complex, which is the key regulator to activate anthocyanin synthesis and accumulation [30,31].

With the development of bioinformatics, the application of transcriptome technology in studies of the flower color of ornamental plants has become mature [32,33,34]. In this study, *N. alata* with white, purple, red and lemon green colors, respectively, were used as materials to analyze the differences of gene expression among plants with different flower colors by transcriptome analyses. These findings could provide a genetic basis for the mechanisms of flower color formation and genetic improvement of *N. alata*.

## 2. Materials and Methods

### 2.1. Plant Materials

**Figure 1:**
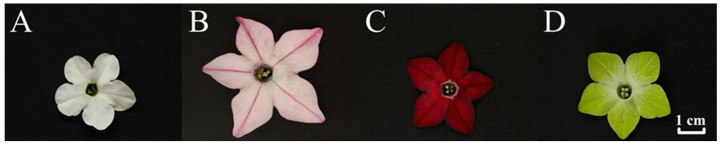
Four colors corollas of *Nicotiana alata*. (**A**) white, (**B**) purple, (**C**) red, (**D**) lemon green.

Four *N. alata* cultivars with different colors provided by the Tobacco Lab of Shandong Agricultural University, including white (signed as W), purple (P), red (R), and lemon green (L), were used as materials (Figure 1), and were grown in the greenhouse (36°20′ N, 117°12′ E). The corollas (CO) of completely opened flowers were selected as samples. The samples were signed as color combined with part. For example, the white corolla was signed as W_CO. These tissues were quickly put into sterile centrifugal tubes, placed in liquid nitrogen for immediately freezing, and then stored at −80 °C.

### 2.2. RNA Extraction and Sequencing

According to the manufacturer’s instructions, total RNA from the corollas of *N. alata* was extracted using an RNA prep Pure Plant kit (Tiangen Biotech, Beijing, China). DNA digestion was carried out after RNA extraction by DNaseI. RNA quality was determined by examining A260/A280 with Nanodrop^TM^ OneCspectrophotometer (Thermo Fisher Scientific Inc., Waltham, MA, USA). RNA integrity was confirmed by 1.5% agarose gel electrophoresis. Qualified RNAs were finally quantified by Qubit3.0 with Qubit^TM^ RNA Broad Range Assay Kit (Q10210, Life Technologies, Ltd., Paisley, UK).

A total of 2 μg total RNAs were used for stranded RNA sequencing library preparation using KC^TM^ Stranded mRNA Library Prep Kit for Illumina^®^ (Catalog NO. DR08402, Seqhealth Co., Ltd., Wuhan, China) following the manufacturer’s instructions. PCR products corresponding to 200–500 bps were enriched, quantified, and finally sequenced on Illumina Novaseq 6000 sequencer (Illumina Inc., San Diego, CA, USA) with a pair-end 150 bp (PE150) model at Seqhealth Technology Co., LTD (Wuhan, China). Three biological replicates were prepared for each tissue.

### 2.3. De Novo Assembly and Differential Expression Genes (DEGs) Analysis

The adapter sequences and unknown or low-quality reads were filtered by Trimmomatic (v0.39) [35] with default options to obtain high-quality reads. FastQC (v0.11.9, http://www.bioinformatics.babraham.ac.uk/projects/fastqc/, accessed on 15 September 2020) was used to assess the quality of reads. The high-quality reads of the corollas were assembled de novo by Trinity (v2.1.1) [36] using default settings, and the set of non-redundant unigenes was obtained by TGICL (v2.1) [37] and Cd-Hit (v4.8.1) [38]. To identify transcripts corresponding to putatively full-length transcripts spanning protein-coding sequences (CDS), BlastN (v2.11.0, https://blast.ncbi.nlm.nih.gov/Blast.cgi, accessed on 3 March 2021) search results of transcripts against CDS of close species (*Nicotiana sylvestris*) were used for calculation coverage of transcripts on CDS of *Nicotiana sylvestris* with the highest bit score. Full-length transcripts were defined as transcripts with at least 90% coverage on matched CDS and with a length over 300 bp [39].

The gene expression level was calculated using the Fragments Per Kilobase of exon per Million fragments mapped (FPKM) method by the RSEM (v1.3.3) [40] with default parameters. The expected counts of reads were also calculated. DEGs were identified by RSEM (command: rsem-run-ebseq) with the false discovery rate (FDR) <0.05 and |log2 (fold change)| > 1. The expression of DEGs was significantly different when |log2 (fold change)| > 2.

### 2.4. Functional Annotation and Enrichment Analysis

Unigenes were annotated by BlastX searches against the Swiss-Prot protein database (http://www.expasy.ch/sprot, accessed on 22 March 2021) with *E*-value ≤ 10^−5^. Only the top hit for each sequence was extracted. According to the homology annotations against the Swiss-Prot protein database, gene ontology (GO) annotations of unigenes were obtained by TBtools (v1.09852) [37]. To obtain the metabolic pathway annotation, the nucleotide sequences of unigenes were aligned to the Kyoto Encyclopedia of Genes and Genomes Pathway (KEGG) database on the KEGG Automatic Annotation Server (KAAS, https://www.genome.jp/tools/kaas/, accessed on 6 April 2021) by the bi-directional best hit (BBH) method, compared with *Arabidopsis thaliana*, *Solanum lycopersicum*, *Solanum pennellii*, *Nicotiana tabacum*, *Nicotiana tomentosiformis*. GO classifications and KEGG pathway enrichment analyses of DEGs were performed using TBtools with a *p*-value < 0.05.

### 2.5. TFs and Functionality Identification

The sequences of TFs related to flower color formation were downloaded from NCBI (Table S1) and were aligned with DEGs by BlastX. To analyze the structures of TFs related to flower color formation, conserved motifs were characterized using Multiple Em for Motif Elicitation (MEME, http://meme-suite.org/tools/meme, accessed on 23 July 2021) with the following parameters: any repetitions; the maximum number of motifs being 5; and 6–50 residues’ width of each motif. The protein interaction networks of DEGs homologous to the genes related to flower color formation were analyzed by protein–protein interaction analysis using the online program STRING (https://version-11-0b.string-db.org, accessed on 5 August 2021) [41]. The Markov Cluster Algorithm (MCL) method was used for clustering in the protein interaction networks.

## 3. Results

### 3.1. RNA-Seq, Sequence Assembly and Differential Expression of Genes

RNA-seq was performed on the corollas of *N. alata* with white, purple, red, and lemon green. A total of 91.8 Gb of clean reads were obtained after sequencing and filtering with Q30 ≥ 97.15% and GC contents of 41.5–43%, which indicated the sequences’ qualities were sufficient for subsequent assembly and analyses (Table S2). A total of 333,046 unigenes were assembled based on clean reads with the N50 of 1623 bp, and the average length of unigenes was 941.81 bp. The full-length transcript covered 40.22% CDS of *Nicotiana sylvestris* (the close species of *N. alata*). A total of 33,445 DEGs were identified between W_CO, P_CO, R_CO and L_CO based on normalized expected_count (Table S3).

### 3.2. Annotation of Unigenes and Functional Enrichment Analysis of DEGs

**Figure 2:**
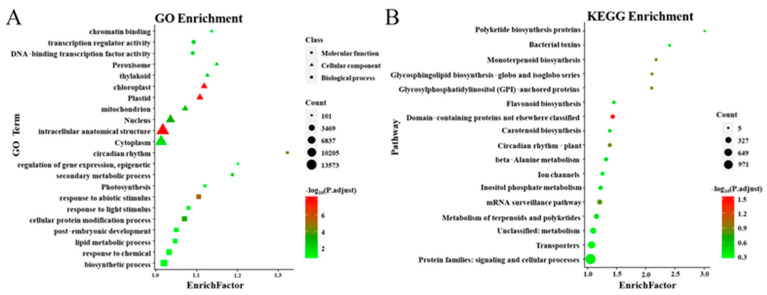
Gene functional enrichment of DEGs. (**A**) GO functional enrichment, (**B**) KEGG pathway enrichment. Rich Factor: represents the degree of enrichment, the higher the value, the higher the enrichment degree; −log_10_ (P. adjust): Benjamini and Hochberg (BH) method was used to obtain the corrected *p*-value, represented by color, red indicates a smaller *q*-value, and indicates more obvious enrichment; Count: expressed as the size of the point, the larger the point said more genes.

A total of 118,541 unigenes (about 35.59% of total unigenes) were annotated to the Swiss-Prot database (Table S3). A total of 97,118 unigenes (about 29.16% of total unigenes and 81.93% of the genes annotated to the Swiss-Prot database) were annotated to the GO database. The enrichment of transcription regulator activity and DNA-binding transcription factor activity indicated that TFs in DEGs played essential roles during flower pigments biosynthesis. Chloroplast” and plastid were enriched, which showed that DEGs were involved in chlorophyll metabolism (Figure 2A and Tables S4 and S5). A total of 39,655 unigenes (about 11.91% of total unigenes) were annotated to the KEGG database and assigned to 239 pathways. Flavonoid biosynthesis, Anthocyanin biosynthesis, and Porphyrin and chlorophyll metabolism, which were concerned with the color formation, were annotated (Figure 2B and Tables S6 and S7).

### 3.3. DEGs Involved in the Chlorophyll Metabolism and Anthocyanin Biosynthesis Pathways

**Figure 3:**
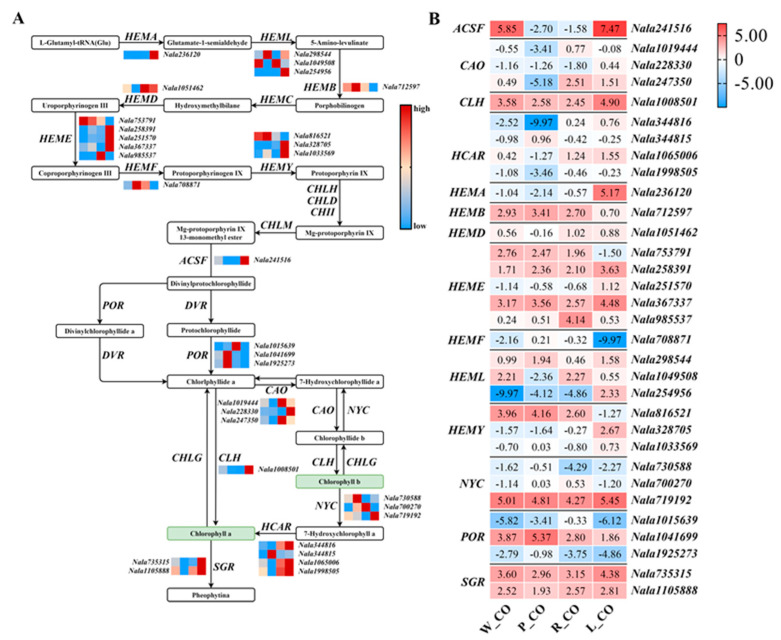
Expression of DEGs in the chlorophyll metabolism pathway of *Nicotiana alata*. (**A**) the chlorophyll metabolism pathway. The heatmap columns are W_CO, P_CO, R_CO, and L_CO from left to right. The red color indicates high expression, and blue indicates low expression. The color change in this part only represents the differential expression of each line of each corolla, and there is no comparability between different lines. (**B**) the expression level of DEGs participated in the chlorophyll metabolism pathway. The heatmap columns are W_CO, P_CO, R_CO, and L_CO from left to right. The number in the cell indicates log2 (FPKM + 0.001). Abbreviations: *HEMA*: glutamyl-tRNA reductase; *HEML*: glutamate-1-semialdehyde 2,1-aminomutase; *HEMB*: 5-aminolevulinate dehydrogenase; *HEMC*: porphobilinogen deaminase; *HEMD*: uroporphyrinogen III synthase; *HEME*: uroporphyrinogen III decarboxylase; *HEMF*: coproporphyrinogen III oxidase; *HEMY*: protoporphyrinogen/coproporphyrinogen III oxidase; *CHLH/D/I*: Mg-chelatase subunit H/D/I; *CHLM*: Mg-protoporphyrin O-methyltransferase; *ACSF*: Mg-protoporphyrin IX monomethylester cyclase; *POR*: protochlorophyllide reductase; *DVR*: divinyl chlorophyllide a 8-vinyl-reductase; *CAO*: chlorophyllide a oxygenase; *NYC*: chlorophyll b reductase; *CLH*: chlorophyllase; *CHLG*: chlorophyll/bacteriochlorophyll a synthase; *HCAR*: 7-hydroxymethyl chlorophyll a reductase; *SGR*: Mg dechelatase.

A total of 32 homologous genes in DEGs were related to the chlorophyll metabolism pathway (Figure 3A). In this pathway, compared with W_CO, P_CO, and R_CO, the expression of *HEMA*, *ACSF*, *CLH* and *SGR* homologues were up-regulated, and that of *HEMB*, *HEMF* and *POR* homologues were down-regulated in L_CO (Figure 3B). Among these, the expression of *HEMA* homologues was significantly up-regulated (more than 53-fold). In contrast with W_CO, 68% homologous DEGs involved in chlorophyll biosynthesis were up-regulated (Figure 3) in L_CO. Compared to W_CO, homologues of *ACSF* and *CLH* were down-regulated, and *HEMF* homologues were up-regulated in P_CO and R_CO. In particular, *ACSF* homologues were significantly down-regulated (more than 172-fold) in this comparison. Homologues of *HEMB*, *HEMF* and *CLH* were up-regulated and *HEMA*, *HEMD*, *ACSF* and *SGR* homologues were down-regulated in the comparison of P_CO vs. R_CO. However, the expression of homologues in this comparison was not significantly different (Figure 3B).

**Figure 4:**
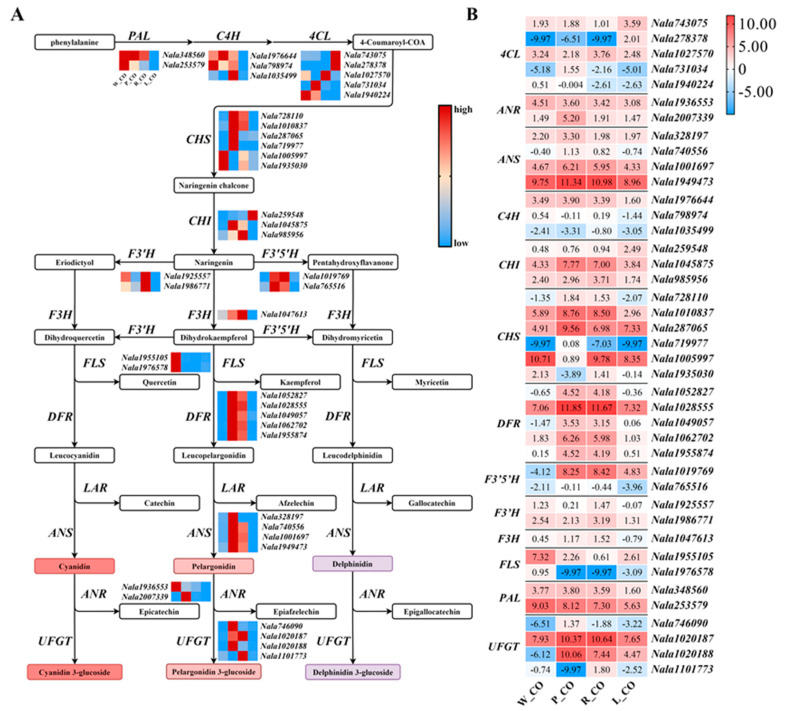
Expression of DEGs in the anthocyanin biosynthesis pathway of *Nicotiana alata*. (**A**) the anthocyanin biosynthesis pathway. The heatmap columns are W_CO, P_CO, R_CO, and L_CO from left to right. The red color indicates high expression, and blue indicates low expression. The color change in this part only represents the differential expression of each line of each corolla, and there is no comparability between different lines. (**B**) the expression level of DEGs participated in the anthocyanin biosynthesis pathway. The heatmap columns are W_CO, P_CO, R_CO, and L_CO from left to right. The number in the cell indicates log2 (FPKM + 0.001). Abbreviations: *PAL*: phenylalanine ammonia-lyase; *C4H*: cinnamate 4-hydroxylase; *4CL*: 4-coumarate-CoA ligase; *CHS*: chalcone synthase; *CHI*: chalcone isomerase; *F3H*: flavanone 3-hydroxylase; *F3’H*: flavonoid 3’-hydroxylase; *F3’5’H*: flavonoid 3’,5’-hydroxylase; *FLS*: flavonol synthase; *DFR*: dihydroflavonol 4-reductase; *ANS*: anthocyanidin synthase; *ANR*: Anthocyanidin reductase; *UFGT*: UDP-glucose:flavonoid 3-O-glucosyltransferase.

A total of 41 homologous genes in DEGs relevant to the anthocyanin biosynthesis pathway were identified in *N. alata* (Figure 4A). Compared to W_CO and L_CO, the expression of DEGs homologous to *F3H*, *F3’5’H* and *DFR* were up-regulated in P_CO and R_CO (Figure 4B). Among these, homologues of *DFR* were significantly up-regulated (more than eight-fold). In the comparison of L_CO vs. W_CO, DEGs homologous of *PAL*, *C4H*, *F3H*, *F3’H*, *FLS*, *ANS,* and *ANR* were down-regulated, especially the homologues of *PAL* and *FLS*, which were significantly differently expressed (more than four-fold and 16-fold, respectively) (Figure 4B). Most structural genes that promote anthocyanin accumulation showed higher expression levels in P_CO and R_CO rather than W_CO and L_CO (Figure 4), consistent with the phenotype of *N. alata*. Compared with others, W_CO showed the highest expression of some early genes that promote anthocyanin accumulation, such as *PAL* (*Nala253579*), but the higher expression of late gene *FLS* (*Nala1976578*), which reduced the accumulation (Figure 4B). In the comparison of P_CO vs. R_CO, homologues of *PAL*, *DFR*, *ANS* and *ANR* were up-regulated, and homologues of *F3’H* and *F3H* were down-regulated. Nevertheless, the expression difference of these DEGs was not significant, except *ANR* homologue (*Nala2007339*) (more than nine-fold).

### 3.4. Analysis of Differential Expression of Tfs and Protein Interaction Networks of the Anthocyanin Biosynthesis Pathways

**Figure 5:**
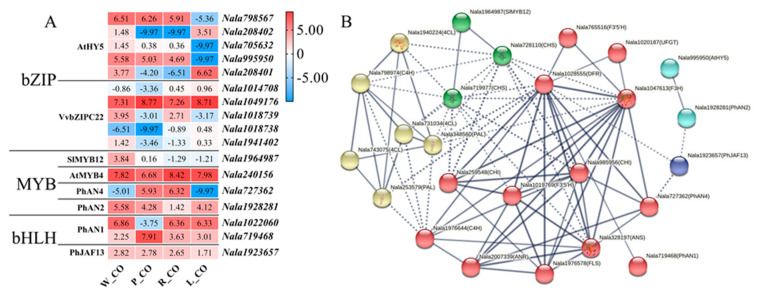
(**A**) the expression level of DEGs in bZIP family, MYB family and bHLH family. The heatmap columns are W_CO, P_CO, R_CO, and L_CO from left to right. The red color indicates high expression, blue indicates low expression, and the number in the cell indicates log2 (FPKM + 0.001). (**B**) analysis of protein interaction networks of TFs and structural genes homologues in DEGs. Different colors represent different clusters, solid lines represent connections among that same cluster, dashed lines represent connections among different clusters, and the thickness of lines represents confidence. The confidence level of this network is >0.7. For clustering units and more details, see Table S8.

To study the regulatory effect of TFs on the anthocyanin biosynthesis pathway, the DEGs homologous to some anthocyanin-related TFs were screened, and then the protein interaction networks of DEGs related to anthocyanin biosynthesis were analyzed. A total of 17 DEGs homologous to TFs related to anthocyanin synthesis were identified, of which 10 were homologous to the bZIP family, 4 to the MYB family, and 3 to the bHLH family (Figure 5A). Analysis of TFs’ structure confirmed that TFs in the same family have similar motifs (Figure S1).

Analysis of protein interaction networks of DEGs, which were homologous to genes which encode TFs and structural enzymes in the anthocyanin biosynthesis pathway, showed that homologues of *AtHY5* (*Nala995950*), *PhAN4* (*Nala727362*), *PhAN2* (*Nala1928281*), *SlMYB12* (*Nala1964987*), *PhAN1* (*Nala719468*) and *PhJAF13* (*Nala1923657*) were involved in the transcription regulation of the anthocyanin biosynthesis pathway (Figure 5B). *AtHY5* homologue (*Nala995950*) was the highest in W_CO, followed by P_CO and R_CO, but none in L_CO. The homologue of *SlMYB12* (*Nala1964987*) was also the highest in W_CO. And *CHS* homologues (*Nala728110* and *Nala719977*) were considered to interact with *SlMYB12* homologue (*Nala1964987*). The expression level of *PhAN4* homologue (*Nala727362*) was the highest in R_CO, followed by P_CO, and hardly expressed in W_CO and L_CO. *PhAN1* homologue (*Nala719468*) had the highest expression level in P_CO, followed by R_CO (Figure 5A).

## 4. Discussion

Flower color is an essential character of plants. Bright flower color is favorable to attract pollinators [42,43], which is of great significance to the reproduction of insect-pollinated plants. Bright flower color is also a vital ornamental character. *N. alata* is an ornamental plant with bright flower colors and a long flowering period, and is mainly pollinated by hawkmoths [44]. However, the flower color formation mechanism of *N. alata* has rarely been studied. In this study, flower transcriptome analyses of four *N. alata* cultivars with different colors were conducted to explore the reasons for differences in flower color. Gene expression differences in the chlorophyll biosynthetic pathway and anthocyanin biosynthetic pathway were found to play important roles in flower color differences of *N. alata* (Figure 2B and Tables S6 and S7).

DFR is a key enzyme in anthocyanin biosynthesis. DFR competes with FLS for substrates to produce leucoanthocyanins [14], which directs the biosynthesis of anthocyanins and flavonols, respectively, to determine the red or white color of flowers [45]. Compared with W_CO, *DFR* was significantly up-regulated, while *FLS* was significantly down-regulated in P_CO and R_CO (Figure 4B), which was considered to be the efficient cause of the color difference between white and purple or red *N. alata*, according to the difference of the expression levels of *DFR* and *FLS* in R_CO, P_CO and W_CO (Figure 4). In the comparison of P_CO and R_CO, the expression of almost all DEGs except *ANR* showed no significant difference (Figure 4). Anthocyanins could show different colors by late diversity modifications, such as polyacylation [46]. Therefore, the difference between purple and red might be due to differences in late anthocyanin modification, such as methylation or acylation in the late stage. In contrast with W_CO, the green color of L_CO might be attributed to the expression of 68% up-regulated DEGs involved in chlorophyll biosynthesis (Figure 3B), especially *HEMA*, a key gene that regulates the chlorophyll synthesis pathway. The expression of DEGs involved in anthocyanin biosynthesis was lower in L_CO (Figure 4B), which made green more conspicuous.

In this research, the presumptive TFs have similar motifs to TFs in other species, which indicates that these have similar functions to TFs in other species (Figure S1). HY5 was believed to induce the expression of *CHS*, *CHI* and other anthocyanin biosynthesis early enzymes [47]. In L_CO, *HY5* (*Nala995950*) was no-expression (Figure 5A). That might be the reason for the low expression of DEGs homologous to anthocyanin biosynthesis early genes in L_CO, which could reduce anthocyanin accumulation in lemon green flowers. MYB12 induces the expression of *CHS* and *FLS* [28]. The expression of *MYB12* (*Nala1964987*) was the highest in W_CO (Figure 5A), leading to the high expression of *CHS* and *FLS* in white flowers (Figure 4B). FLS makes the dihydroflavonol generated in white flowers enter the flavonol biosynthesis pathway instead of the anthocyanin biosynthesis pathway, which reduces anthocyanin accumulation in white flowers. *PhAN1* (*Nala719468*) and *PhAN4* (*Nala727362*) showed higher expression levels in P_CO and R_CO (Figure 5A), and they were thought to activate *DFR* expression [29]. That suggested they promoted anthocyanin accumulation in *N. alata* by up-regulating the expression of *DFR* (*Nala1028555*) (Figure 4B). VvbZIPC22 was reported to regulate anthocyanin biosynthesis by directly or indirectly activating the promoter of enzyme genes in the anthocyanin biosynthesis pathway, such as *CHS*, *CHI*, *FLS* and *ANR* [48]. *VvbZIPC22* (*Nala1049176*) was indeed highly expressed in four corolla samples, but protein interaction networks analysis did not reveal how it was involved in the anthocyanin biosynthesis pathway (Figure 5B). It was believed to play an indirect role in anthocyanin biosynthesis in *N. alata*.

In this study, transcriptome technology was used to explore the reason for the differences in flower color of *N. alata*. Ninety candidate genes related to flower colors were identified. Gene expression differences in chlorophyll and anthocyanin metabolic pathways were considered to be an important reason for the different flower color formations in *N. alata*. This study showed that *DFR* (*Nala1028555*) and *FLS* (*Nala1976578*) played important roles in the biosynthesis of anthocyanins. The regulatory effects of the coding products of *HY5* (*Nala995950*), *MYB12* (*Nala1964987*), *AN1* (*Nala719468*), and *AN4* (*Nala727362*) were also important in the flower color differences of *N. alata*. These findings could provide a genetic basis for further molecular verification of flower color genes, directional improvement, and epigenetic research on flower color in *N. alata*.

## Data Availability

The sequencing data for the clean reads were deposited in the National Center for Biotechnology Information (NCBI) Sequence Read Archive (http://www.ncbi.nlm.nih.gov/sra, accessed on 18 November 2021) with accession number SRR16966198-SRR16966209.

## Supplementary Materials

The supplementary materials are available online at https://www.mdpi.com/article/10.3390/genes12121976/s1. Table S1. TFs associated with anthocyanin biosynthesis from the NCBI. Table S2. Data qualities statistics of RNA-seq in *Nicotiana alata*. Table S3. All DEGs with annotations. Table S4. Results of all GO enriched. Table S5. Results of GO enriched with a *p*-value < 0.05. Table S6. Results of all KEGG enriched. Table S7. Results of KEGG enriched with a *p*-value < 0.05. Table S8. Clusters of Protein interaction network. Figure S1. Motifs distribution of DEGs and homologues in bZIP family, MYB family and bHLH family.
