# Supplementary material for: Important Roles of Key Genes and Transcription Factors in Flower Color Differences of Nicotiana alata
Source: Genes (Basel). 2021 Dec 10;12(12):1976. doi: 10.3390/genes12121976 (PMC8701347; doi:10.3390/genes12121976)
Supplement: Supplementary file 1 [file genes-12-01976-s001.zip › Table S2.pdf]

genes

MDPI

Table S2. Data qualities statistics of RNA-seq in flowering tobacco.

Sample	raw_read	clean_read	raw_base(G)	clean_base(G)	Q20(%)	Q30(%)	Deduplicated_ Percentage	GC(%)
L-CO-1	36744742	34280740	5.51	4.98	100	97.5	36.55	41.5
L-CO-2	52874984	48737460	7.93	7.06	100	97.8	35.85	41.5
L-CO-3	39866368	37031896	5.98	5.36	100	97.35	41.35	41.5
P-CO-1	36834678	34348378	5.53	5	100	97.6	40.65	42
P-CO-2	36236828	33450040	5.44	4.86	100	97.55	40.85	42
P-CO-3	58114286	53281472	8.72	7.73	100	97.95	36.85	42
R-CO-1	59998690	54475584	9	8.01	100	99.1	34.35	42.5
R-CO-2	62766918	56661046	9.42	8.33	100	99.1	32.75	43
R-CO-3	67180028	57199086	10.08	8.39	100	99	35.9	42.5
W-CO-1	37929434	34730542	5.69	5.09	100	98.25	36.85	42.5
W-CO-2	42030266	39289096	6.3	5.75	100	98.15	39.5	41.5
W-CO-3	39340808	36359010	5.9	5.33	100	98.25	37.1	42.5
