# Supplementary material for: Important Roles of Key Genes and Transcription Factors in Flower Color Differences of Nicotiana alata
Source: Genes (Basel). 2021 Dec 10;12(12):1976. doi: 10.3390/genes12121976 (PMC8701347; doi:10.3390/genes12121976)
Supplement: Supplementary file 1 [file genes-12-01976-s001.zip › Table S1.pdf]

genes

MDPI

Table S1. TFs associated with anthocyanin biosynthesis from NCBI.

Species	Family	Description	GeneID	GI
Arabidopsis thaliana	bHLH	EGL3	842669	-
Arabidopsis thaliana	bHLH	GL3	834133	-
Arabidopsis thaliana	bHLH	TT8	826571	-
Arabidopsis thaliana	bZIP	HY5	830996	-
Arabidopsis thaliana	bZIP	HYH	821027	-
Arabidopsis thaliana	MYB	CPC	819249	-
Arabidopsis thaliana	MYB	MYB4	830018	-
Arabidopsis thaliana	MYB	MYB7	816173	-
Arabidopsis thaliana	MYB	MYB11	825435	-
Arabidopsis thaliana	MYB	MYB12	819359	-
Arabidopsis thaliana	MYB	MYB32	829651	-
Arabidopsis thaliana	MYB	PAP2	842957	-
Arabidopsis thaliana	MYB	MYB111	834993	-
Arabidopsis thaliana	MYB	MYB113	842955	-
Arabidopsis thaliana	MYB	MYB114	842956	-
Arabidopsis thaliana	MYB	PAP1	842120	-
Arabidopsis thaliana	MYB	TT2	833520	-
Arabidopsis thaliana	WD40	TTG1	832523	-
Petunia x hybrida	bHLH	AN1	-	10998406
Petunia x hybrida	bHLH	JAF13	-	3127045
Petunia x hybrida	MYB	AN2	-	673536266
Petunia x hybrida	MYB	AN4	-	311700631
Petunia x hybrida	WD40	AN11	-	2290532
Solanum lycopersicum	MYB	AN2-like	101250648	-
Solanum lycopersicum	MYB	ANT1	543897	-
Solanum lycopersicum	MYB	MYB12	100191123	-
Solanum lycopersicum	MYB	MYBTV	101250818	-
Vitis vinifera	bHLH	MYC1	100251098	-
Vitis vinifera	bZIP	bZIPC22	100257331	-
Vitis vinifera	MYB	MYB5b	100232973	-
Vitis vinifera	MYB	MYB114	100249722	-
Vitis vinifera	MYB	MYBA1	100233098	-
Vitis vinifera	MYB	MYBA6	100243253	-
Vitis vinifera	MYB	MYBA7	100265568	-
Vitis vinifera	MYB	MYBPA1	100232899	-
Vitis vinifera	MYB	TT2	100254224	-
