# Supplementary figures and images for: Important Roles of Key Genes and Transcription Factors in Flower Color Differences of Nicotiana alata
Source: Genes (Basel). 2021 Dec 10;12(12):1976. doi: 10.3390/genes12121976 (PMC8701347; doi:10.3390/genes12121976)

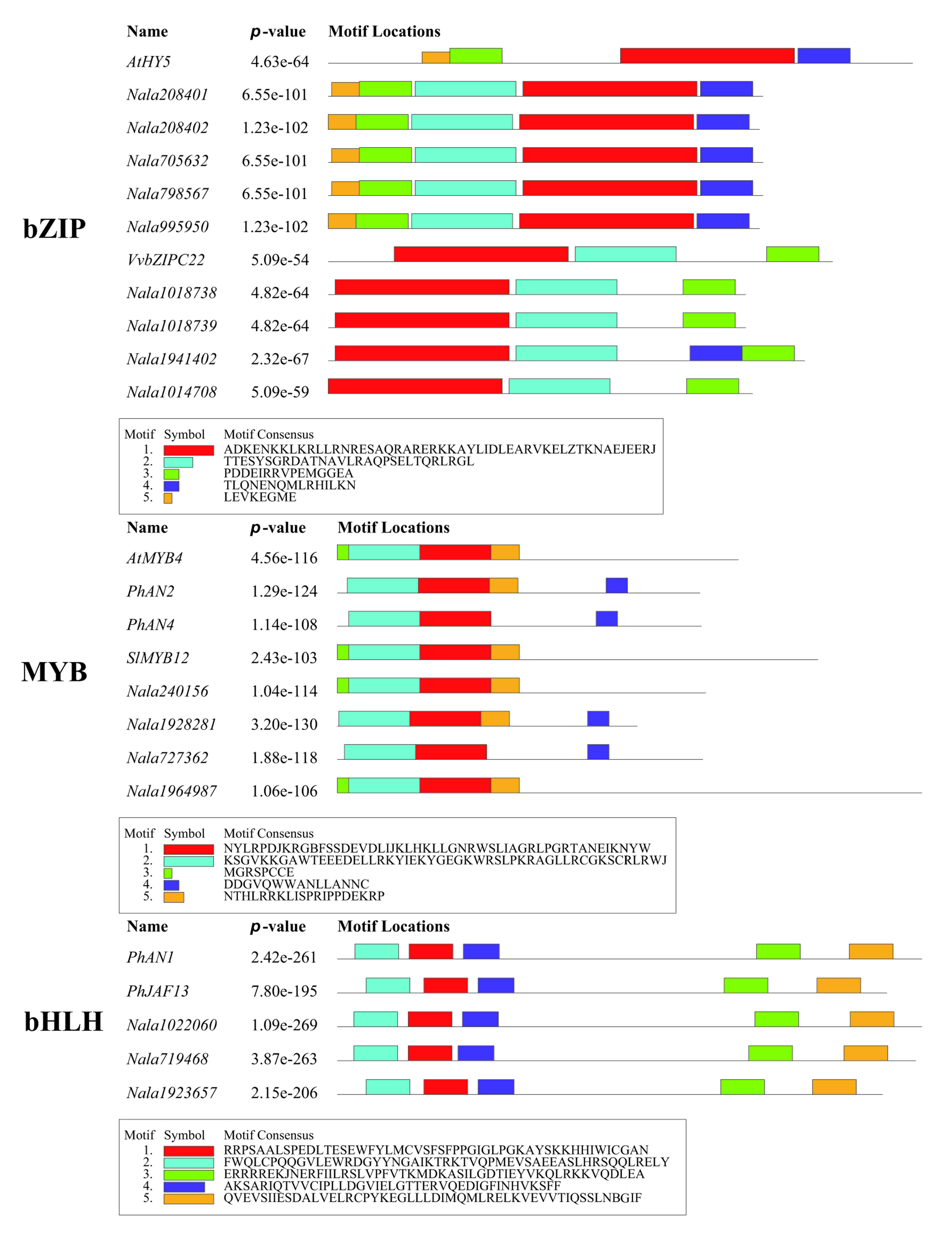

Supplement: Supplementary file 1 [file genes-12-01976-s001.zip › Figure S1.tif]
